# Mortality Prediction Utilizing Blood Biomarkers to Predict the Severity of COVID-19 Using Machine Learning Technique

**DOI:** 10.3390/diagnostics11091582

**Published:** 2021-08-31

**Authors:** Tawsifur Rahman, Fajer A. Al-Ishaq, Fatima S. Al-Mohannadi, Reem S. Mubarak, Maryam H. Al-Hitmi, Khandaker Reajul Islam, Amith Khandakar, Ali Ait Hssain, Somaya Al-Madeed, Susu M. Zughaier, Muhammad E. H. Chowdhury

**Affiliations:** 1 Department of Electrical Engineering, Qatar University, Doha 2713, Qatar; tawsifur.rahman@qu.edu.qa (T.R.); khreaj@gmail.com (K.R.I.); amitk@qu.edu.qa (A.K.); 2 Department of Basic Medical Sciences, College of Medicine, QU Health, Qatar University, Doha 2713, Qatar; fa1512243@qu.edu.qa (F.A.A.-I.); fa1004485@qu.edu.qa (F.S.A.-M.); rm1104195@qu.edu.qa (R.S.M.); ma1514109@qu.edu.qa (M.H.A.-H.); 3 Medical ICU, Hamad General Hospital, Doha 3050, Qatar; a_aithssain@hotmail.com; 4 Department of Computer Science and Engineering, Qatar University, Doha 2713, Qatar; S_alali@qu.edu.qa

**Keywords:** machine learning, D-dimer, biomarkers, COVID-19, coagulopathy

## Abstract

Healthcare researchers have been working on mortality prediction for COVID-19 patients with differing levels of severity. A rapid and reliable clinical evaluation of disease intensity will assist in the allocation and prioritization of mortality mitigation resources. The novelty of the work proposed in this paper is an early prediction model of high mortality risk for both COVID-19 and non-COVID-19 patients, which provides state-of-the-art performance, in an external validation cohort from a different population. Retrospective research was performed on two separate hospital datasets from two different countries for model development and validation. In the first dataset, COVID-19 and non-COVID-19 patients were admitted to the emergency department in Boston (24 March 2020 to 30 April 2020), and in the second dataset, 375 COVID-19 patients were admitted to Tongji Hospital in China (10 January 2020 to 18 February 2020). The key parameters to predict the risk of mortality for COVID-19 and non-COVID-19 patients were identified and a nomogram-based scoring technique was developed using the top-ranked five parameters. Age, Lymphocyte count, D-dimer, CRP, and Creatinine (ALDCC), information acquired at hospital admission, were identified by the logistic regression model as the primary predictors of hospital death. For the development cohort, and internal and external validation cohorts, the area under the curves (AUCs) were 0.987, 0.999, and 0.992, respectively. All the patients are categorized into three groups using ALDCC score and death probability: Low (probability < 5%), Moderate (5% < probability < 50%), and High (probability > 50%) risk groups. The prognostic model, nomogram, and ALDCC score will be able to assist in the early identification of both COVID-19 and non-COVID-19 patients with high mortality risk, helping physicians to improve patient management.

## 1. Introduction

The Coronavirus Disease 2019 (COVID-19) pandemic continues to strike the globe with second and third waves of infections, as the emerging variants of Severe acute respiratory syndrome coronavirus 2 (SARS-CoV-2) are more transmissible and deadly [1]. Different countries are vaccinating their population with several vaccines to reduce the disease burden and mitigate the pandemic, but, in this race, all countries are not at the same level [2,3]. COVID-19 vaccine production, distribution, and administration have not reached the needed vaccine coverage globally [2,3]. Therefore, the spread of emerging SARS-CoV-2 variants is exceeding the speed of vaccination campaigns resulting in a continuous global burden of COVID-19 disease. As of today, 7 August 2021, there have been a total number of approximately 201 million cases worldwide with 4.27 million deaths [4].

COVID-19 has a spectrum of clinical presentations ranging from asymptomatic patients to critically ill patients. According to several studies [5,6,7,8,9], the severity of the disease depends mostly on age and comorbid conditions. Moreover, genetic factors are also being studied to identify the relationship of COVID-19 with severity [10]. In one particular study, a link was found between genes encoding blood groups, specifically type A, with serious clinical manifestations [11,12]. There are many complications associated with COVID-19 and patients may present with symptoms affecting multiple systems including respiratory, cardiovascular, and gastrointestinal (GI), in addition to affecting coagulability [13,14]. COVID-19 is known to affect coagulation profile and cardiac biomarkers. The rates of cardiac injury among COVID-19 patients are between 19.7% and 27.8% of admitted cases and the associated mortality rates are between 23% and 51.2% [15]. An analysis of coagulopathy, inflammation, and troponin can help to explain the mechanism of myocardial injury. Notably, raised troponin levels among critically ill patients point to cardiac injury and are a sign of poor prognosis. It is a clear indication that the cytokine divulgence syndrome potentially mediates myocardial injury. Longitudinal follow-up insinuates a notable divergence between critically ill patients who die and those who do not. Lastly, cardiac injury during admission relates to severe outcomes. On the third day, C-reactive protein (CRP), a blood marker, measures the level of inflammation. CRP is a protein made by the liver and sent into the bloodstream in response to inflammation. Interleukin-6 (IL-6) is also an inflammatory marker, which is an indicator of disease severity [16], and it was found that the IL-6 peak among critically ill survivors falls between the fourth and seventh days [15]. By contrast, this increases continuously among those who do not survive. D-dimer, a marker of coagulopathy, remains high in those who do not survive in contrast to those who do. COVID-19 is also associated with changes in levels of various circulatory inflammatory coagulation biomarkers including fibrinogen and D-dimer. D-dimer levels have been noticed to be within normal ranges or slightly increased in the early stages of the disease. As the disease and severity progress, levels of D-dimer are significantly increased [17]. Fibrinogen, a protein produced by the liver, also increases with inflammation and a coagulation bio-marker. Creatinine, Lactate Dehydrogenase (LDH) levels, Lymphocyte count, D-Dimer, Troponin, IL-6 and CRP are shown to be important biomarkers for the severity prognosis of COVID-19. Creatinine is a chemical compound leftover from energy-producing processes in the muscles, which a healthy kidney filters out of the blood. LDH is an enzyme involved in energy production, which is found in almost all cells in the body, used to monitor tissue damage associated with a wide range of disorders, including liver disease and interstitial lung disease. The increase of LDH reflects tissue damage, which suggests a viral infection or lung damage, such as the pneumonia induced by SARS-CoV-2 [18].

Assessing COVID-19 severity and prognosis has been of great importance in clinical patient management. Machine learning has played a noteworthy role in detecting COVID-19 using clinical data and chest X-ray and computer tomography images in patients [19,20,21,22,23,24,25]. Banerjee et al. in [26] used full blood counts to recognize COVID-positive cases, instead of the traditional identification of symptoms, and have found that positive patients exhibit lower amounts of leukocytes, platelets, and lymphocytes. Brinati et al. [27] used routine blood biomarkers to test a sample of 279 COVID patients using machine learning models, which results in accuracy ranging between 82% and 86%, and sensitivity ranging between 92% and 95%. Yang et al. [28] evaluated the use of machine learning in routine laboratory blood tests to predict COVID-19, which offers an opportunity for early detection of the illness in areas where RT-PCR tests are not available. Machine learning was also used to predict mortality and critical events in patients with COVID-19. Rahman et al. [29] used easily available complete blood count (CBC) parameters to predict the severity of COVID-19 patients and the developed model was validated on another external dataset reporting very high classification accuracy. Chowdhury et al. [24] investigated demographic and clinical characteristics and patient outcomes using machine learning tools to identify key biomarkers in order to predict the mortality of the individual patient. A nomogram was developed for predicting the mortality risk among COVID-19 patients. Lactate dehydrogenase, neutrophils (%), lymphocyte (%), highly sensitive C-reactive protein, and age (LNLCA), information acquired at hospital admission, were identified as key predictors of death by the multi-tree XGBoost model. The area under the curve (AUC) of the nomogram for the derivation and validation cohort was 0.961 and 0.991, respectively. An integrated score was calculated with the corresponding death probability. COVID-19 patients were divided into three subgroups: low-, moderate- and high-risk groups. Vaid et al. in [30] claim that with the XGBoost classifier, such trends as acute kidney injury, elevated LDH, tachypnea, hyperglycemia, higher age, anion gap, and C-reactive protein were the strongest drivers associated with mortality and critical events. Aladag and Atabey [31] have attempted to predict mortality risk for critical COVID-19 patients using coagulopathy markers. Terwangne et al. in [32] showed the predictive accuracy of severity classification of COVID-19 using a model based on Bayesian network analysis with the help of five important parameters: acute kidney injury, age, Lactate Dehydrogenase Levels (LDH), lymphocytes and activated prothrombin time (aPTT).

Huang et al. [5] used nine independent risk factors at admission to the hospital to quantify the risk score and stratify the patients into various risk groups in a retrospective, multicenter analysis of 336 confirmed COVID-19 patients and 139 control patients. This research did not use any external validation. The independent relationship between the baseline level of four indicators (Neutrophil to Lymphocyte Ratio (NLR), LDH, D-dimer, and CT score) on admission and the severity of COVID-19 was assessed using logistic regression. The presence of high levels of NLR and LDH in serum could help in the early detection of COVID-19 patients who are at high risk. It was shown that the usage of LDH and NLR together increased detection sensitivity [6]. This model, however, is based on a CT image-based ranking, which is not available for all patients. In a limited number of hospitalized patients (84) with COVID-19 pneumonia, Liu et al. [7] suggested combining the NLR and CRP to predict 7-day disease severity. A retrospective cohort of 80 COVID-19 patients treated at Beijing You’an Hospital was analyzed to identify risk factors for serious and even fatal pneumonia and establish a scoring system for prediction, which was later validated in a group of 22 COVID-19 patients [8]. Age, diabetes, coronary heart disease (CHD), percentage of lymphocytes (LYM percent), procalcitonin (PCT), serum urea, CRP, and D-dimer were found to be correlated with mortality by LASSO binary logistic regression in a total of 2529 COVID-19 patients. The researchers then used multivariable analysis to determine that old age, CHD, LYM percent, PCT, and D-dimer independently posed risks for mortality. A COVID-19 scoring system (CSS) was developed based on the above variables to classify patients into low-risk and high-risk categories with discrimination of AUC = 0.919 and calibration of *p* = 0.64 [9].

Although there have been recent works utilizing machine learning approaches for early mortality prediction of patients using biomarkers [5,7,8,9,33,34,35,36,37,38], to the best of the authors’ knowledge, there has been no work to develop a generalized and reliable model for both COVID-19 and non-COVID-19 patients, which is the motivation behind this study, and important to develop during the pandemic situation when medical personnel are dealing with both types of patient. It is critical for both resource planning and treatment planning to identify and prioritize the patients at high risk. In addition, it should be possible for high-risk patients to be constantly tracked during their hospital stay using a reliable scoring method. The patients at risk, who typically end with ill outcomes, require treatment in an intensive care unit (ICU), which can be identified by the proposed tool, helping in saving the lives of a significant number of people during this pandemic. Thus, the novelty of the work in this paper can be stated as the development of a generalized and reliable early mortality risk predicting technique for identifying the patients with high risk among both COVID-19 and non-COVID patients. It also adds to the body of knowledge for developing a framework of prognostic models using machine-learning approaches. This paper not only develops a nomogram-based scoring technique but also validates the performance on a completely unseen dataset from different countries and populations.

The rest of the paper is organized as follows: Section 2 discusses the methodology of the study by describing the datasets used in this paper, the details of data pre-processing stages for machine learning classifiers, and the nomogram-based scoring technique. Section 3 discusses the result of the classification models and nomogram-based scoring techniques. Section 4 discusses the result and validates the performance of the developed nomogram-based scoring technique and, finally, the article is concluded in Section 5.

## 2. Methodology

**Figure 1:**
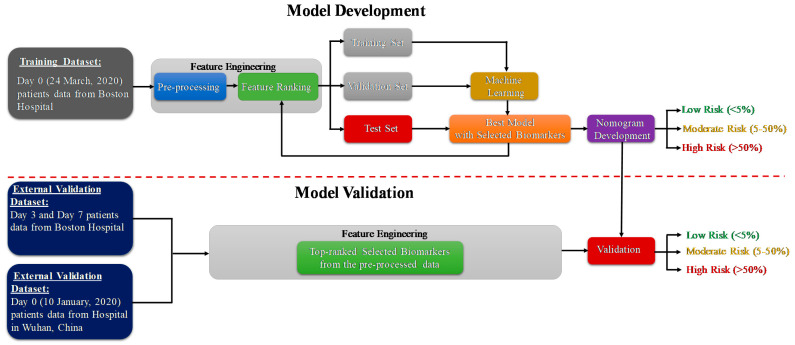
Methodology of the study.

The study consists of two important phases: the model development and model validation phase using two datasets. Dataset-1 [39] (Day-0 patient’s data) is used for the prediction model development and Dataset-2 [40] is used for external validation of the developed model. The code for machine learning pipeline used in this study can be found in [41]. As further illustrated in the methodology diagram (Figure 1), Day-3 and Day-7 patients’ data from dataset 1 is also used for external validation. Pre-processing, and feature selection and reduction were important parts of the feature-engineering task. In the model development phase, the authors have divided the training dataset (Day-0 patients’ data from dataset 1) with the selected features into training, validation and testing data. The validation dataset is used for the tuning of hyper parameters in the machine learning process, and the testing dataset is used for model evaluation. The best-trained model is used to develop the scoring technique to classify the patients into three mortality risk categories: Low, Medium and High. Finally, the developed model is validated using external datasets and the results are reported. The remaining part of the section will provide details of the datasets, pre-processing techniques, performance metrics for machine learning model and the nomogram based scoring technique.

### 2.1. Study Population

In this study, two clinical biomarker datasets from two different countries were used. The first dataset (Dataset-1) was used to develop and validate an early death prediction model and the second dataset (Dataset-2) was used as an external validation model. The first dataset was created from the Emergency Department (ED) of a metropolitan and academic hospital in Boston during the first wave of the COVID-19 pandemic from 24 March 2020 to 30 April 2020. The study was carried out with institutional ethical approval [39]. Patients 18 years or older with clinical concern at the time of hospital admission with acute respiratory illness were included in the study, with at least one of the following conditions: (1) tachypnea (about 22 breaths per minute), (2) oxygen saturation ≤ 92% on room air, (3) supplemental oxygen requirement or (4) positive pressure ventilation requirement. The patients were monitored up to 28 days after registration for the clinical outcome or discharged if the patient recovered. The dataset consists of the biomarkers for three separate days (0, 3, and 7 days). There are six groups of patients available in the enrolled 384 patients. Among them, the first group (Class 1) were the patients with death outcomes (49 (12.76%) patients) and the other groups (Class 2–6) were the patients in the survived class (335 (87.24%) patients). Among the 384 patients, 78 (20%) patients tested as SARS-CoV-2 negative and 306 (80%) patients tested as SARS-CoV-2 positive by RT-PCR. Table 1 shows the description of the first dataset (Dataset-1).

**Figure 2:**
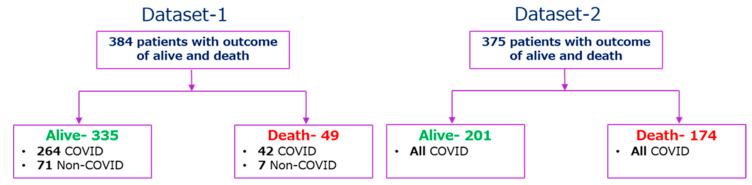
Outcome tree for the patients of Dataset-1 and Dataset-2.

The second dataset (Dataset-2) was collected retrospectively from 375 patients in Wuhan, China between 10 January and 18 February 2020 to find valid and relevant clinical markers of mortality risk. Standard case report forms were used to collect medical records, which included information on epidemiological, demographic, clinical, laboratory, and mortality results. Yan et al. [18] have published the dataset along with their article, and the original study was approved by the Tongji Hospital Ethics Committee. 187 (49.9%) patients had fever symptoms among 375 patients, while cough, weariness, dyspnea, chest discomfort, and muscular pain were reported for 52 (13.9%), 14 (3.7%), 8 (2.1%), 7 (1.9%), and 2 (0.5%) patients, respectively. Among 375 COVID-19 positive patients, 174 and 201 patients were classified as (‘1′) for those who died and (‘0′) for those who survived respectively. Patients’ outcomes with the condition of COVID-19 positive and negative are summarized in Figure 2. There are 76 parameters present in the dataset; the common parameters of Dataset-1, 2 were used for this study, and the parameters from Dataset-2 were normalized in the same way as they appear in Dataset-1, as shown in Table 1, so that Dataset-2 can be used as an external validation set.

### 2.2. Statistical Analysis

Python 3.7 and Stata/MP 13.0 were used to conduct the statistical analysis. Continuous variables, age, and other biomarkers were reported with the number of missing data, and frequency for each biomarker in death and survival groups. Chi-square univariate test was conducted to identify the statistically significant different features among the dead and survived group and the difference is considered significant if the *p*-value is <0.05. There were 20 features present in the original dataset; the top five features using the feature selection method were identified as promising (reported in the later section) and are summarized in Table 2A,B for Dataset-1, and Dataset-2, respectively. The ranked five features were Age, Lymphocyte count, D-dimer, Creatinine, and CRP.

### 2.3. Data Preprocessing

While patient’s blood sample data were available for multiple days, the study used first-day data (from Dataset-1) for model training and validation to identify the primary predictors of the severity of the disease. The model also helps to differentiate between patients who need urgent medical support. Clinical data always suffer from missing data problems that contribute to either biased models or degradation in model performance. This problem can be tackled by deleting the corresponding rows of data for further investigation, but it is stated in [38] that this easy method of removing missing data rows can often lead to the loss of important data that would have been useful in the study, and can also lead to skewed estimates. To fix the missing data, many standard data imputation techniques are available. The most common technique for clinical data imputation is multiple imputations using the chained equations (MICE) data imputation technique [42]. Based on the other variables present in the dataset, the missing data is estimated using multiple regression models. The technique often takes into account the data form of the missing variables before imputing them. Using logistic regression, binary variables are predicted, while continuous variables are predicted using statistical mean matching [38]. Supplementary Figure S1 shows the number of missing values in different features in Dataset-1. Most of the features appear to be completely populated, while Lymphocyte count, d-dimer, creatinine, LDH, monocyte, CRP, and neutrophils seem spottier. The spark-line at right summarizes the general shape of the data completeness in the dataset. The imbalanced data can result in a biased model and, therefore, the dataset needs to be balanced. The synthetic minority oversampling technique (SMOTE) is a powerful approach to tackle the imbalance problem [43]. In this study, alive patients are about seven times more frequent than dead patients, so SMOTE was used for balancing the data.

Twenty different features present in Dataset-1 were checked to identify the correlation among different features. Feature reduction, with the help of the removal of highly correlated features, has always helped in improving the classifier performance [44]. Supplementary Figure S2 shows the heat map of correlation and it is found that most of them are not correlated with each other. The maximum correlation found between creatinine and kidney parameters is 0.56. Therefore, no feature can be removed based on correlation; rather feature ranking and identifying the best feature combination for stratifying the dead and survived group is required.

### 2.4. Development and Validation of Classification Model

The authors have investigated different machine learning classifiers: Random Forest [45], Support Vector Machine (SVM) [46], K-nearest neighbor (KNN) [47], XGBoost [48], Extra-tree [49] and Logistic regression [50]. Logistic regression was the best performing machine learning classifier and has been used in this study (Table 3). Logistic Regression is also a commonly used model for clinical investigation and is a supervised machine learning method for classification tasks [50]. When we want to estimate the likelihood of a binary classification problem (i.e., survival or death of a patient), this technique is very popular [51]. The logistic function is a sigmoid function and shrinks continuous inputs into a probability value. The logistic regression classifier is used to classify the data into two classes: Death and Survived using the ranked features, and the best feature combination is identified for both COVID and NON-COVID data and COVID data alone.

Dataset-1 was divided into training and validation sets (80% of the data) and testing sets (20%). Different machine learning models were investigated using five-fold cross-validation. The performance of different models was evaluated on the test dataset using several performance metrics, including sensitivity, specificity, precision, accuracy, and F1-score as shown in Equations (1)–(5). The receiver operating characteristic curve, or ROC curve, is used to measure the area under the curve (AUC) separately for single predictors as well as for a combination of them. To determine the performance of various top-ranked parameters in stratifying dead and survived patients, the AUC values for different individual features and their combinations’ contributions were evaluated. The performance of unseen (test) folds was combined to create the overall confusion matrix for the five-fold.

(1) $$\mathrm{Accuracy}=\frac{\left(\mathrm{TP}+\mathrm{TN}\right)}{\left(\mathrm{TP}+\mathrm{FN}\right)+\left(\mathrm{FP}+\mathrm{TN}\right)}.$$

(2) $$\mathrm{Sensitivity}=\frac{\left(\mathrm{TP}\right)}{\left(\mathrm{TP}+\mathrm{FN}\right)}.$$

(3) $$\mathrm{Specificity}=\frac{\left(\mathrm{TN}\right)}{\left(\mathrm{FP}+\mathrm{TN}\right)}.$$

(4) $$\mathrm{Precision}=\frac{\left(\mathrm{TP}\right)}{\left(\mathrm{TP}+\mathrm{FP}\right)}.$$

(5) $$F1\;\mathrm{Score}=\frac{\left(2\times \mathrm{TP}\right)}{\left(2\times \mathrm{TP}+\mathrm{FN}+\mathrm{FP}\right)}.$$

The number of patients with death outcome classified as death, the number of survived patients identified as survivors, the number of survived patients incorrectly identified as death, and the number of death patients incorrectly identified as survivors, respectively, are denoted by the true positive (TP), true negative (TN), false positive (FP), and false-negative (FN).

### 2.5. Development and Validation of Logistic Regression-Based Nomogram

The study proposed a diagnosis nomogram based on multivariate logistic regression analysis and Stata/MP software version 13.0, which was developed using Alexander Zlotnik’s Nomolog [52]. Nomograms are graphic representations of complicated mathematical formulas. Medical nomograms graphically represent a statistical prognostic model that predicts a likelihood of a clinical event, such as cancer recurrence or death, for a specific individual, using biologic and clinical data such as tumor grade and patient age. Each variable is listed separately in a nomogram, with a corresponding number of points allocated to each variable’s magnitude. The total point score for all factors is then matched to an outcome scale [53]. A binary regression is used in logistic (logit) regression to estimate the parameter. The dependent variable, generally labeled ‘0′ and ‘1′, is the response variable. Those that survived are marked with a ‘0′, while those who died are marked with a ‘1′. Equation (6) shows the odds, which shows the ratio of probability (Pr) of occurring death and not occurring death (1 − Pr). While the probability can vary from 0 to 1, the odds can vary from 0 to ∞. The logarithm of odds is a linear combination of one or more independent variables (predictors) in the logistic regression. The independent variables can be a binary variable (e.g., gender) and a continuous variable (e.g., age). The log of odds can be termed as linear prediction (LP), as seen in Equation (7), and can be related to the probability of a particular outcome (e.g., death). Equations (6)–(9) are used to create a relationship between death probability and the key predictors using logistic regression.

(6) $$\mathrm{odds}=\frac{\mathrm{Pr}}{1-\;\mathrm{Pr}}.$$

(7) $$\mathrm{Linear}\;\mathrm{Prediction}=\ln\left(\mathrm{odds}\right)=\ln\left(\frac{\mathrm{Pr}}{1-\;\mathrm{Pr}}\right)\;=b_{0}+b_{1}x_{1}+b_{2}x_{2}+\cdots +b_{n}x_{n}.$$

(8) $$\frac{\mathrm{Pr}}{1-\;\mathrm{Pr}}=e^{b_{0}+b_{1}x_{1}+b_{2}x_{2}+\cdots +b_{n}x_{n}}=e^{\mathrm{Linear}\;\mathrm{Prediction}}.$$

(9) $$P=\frac{e^{\mathrm{Linear}\;\mathrm{Prediction}}}{1+e^{\mathrm{Linear}\;\mathrm{Prediction}}}=\frac{1}{1+e^{-\mathrm{Linear}\;\mathrm{Prediction}}}.$$

The logistic regression-based nomogram was created using the top-ranked independent variables with the best performance. The clinical parameters from Dataset-1′s Day-0 data were utilized for model creation, while day-3, day-7, and data upon hospital admission from Dataset-2 were used for model validation. Internal calibration curves, with the first dataset, and external calibration curves, with the second dataset, are used to compare the performance of the developed model. To determine the threshold values at which nomograms will be clinically relevant, the study used Decision Curve Analysis (DCA).

### 2.6. Nomogram-Based Scoring System

Nomograms are widely used for clinical prognosis as they can help in simplifying statistical predictive models into probability of an event, i.e., mortality in this study. They are preferred by clinicians due to their user-friendly graphical interfaces [54]. The nomogram represents many independent variables as a numerated horizontal axis scale, with the patient’s values placed on that scale. From the many parameters numerated and arranged scales, a vertical line was traced down to a horizontal score axis. On the score axis, all of the scores from the independent variables were combined to create a total score, which was then linked to a death probability, which was a horizontal axis scaled from 0 to 1. It should be emphasized that, according to the nomogram, a greater score indicates a higher risk of mortality. The model was created using the patients’ Day-0 data. However, it can be used to longitudinally validate the model to predict death probability using biomarkers acquired later during the patients’ hospital stay.

## 3. Results

This section discusses the following results: (i) identification of the best feature combinations using logistic regression classifier, (ii) developed and validation of the proposed nomogram-based model in predicting death outcomes for the best feature combinations, and finally (iii) a detailed prognostic evaluation of the nomogram.

### 3.1. Univariate and Multivariate Analysis Using Logistic Regression

**Figure 3:**
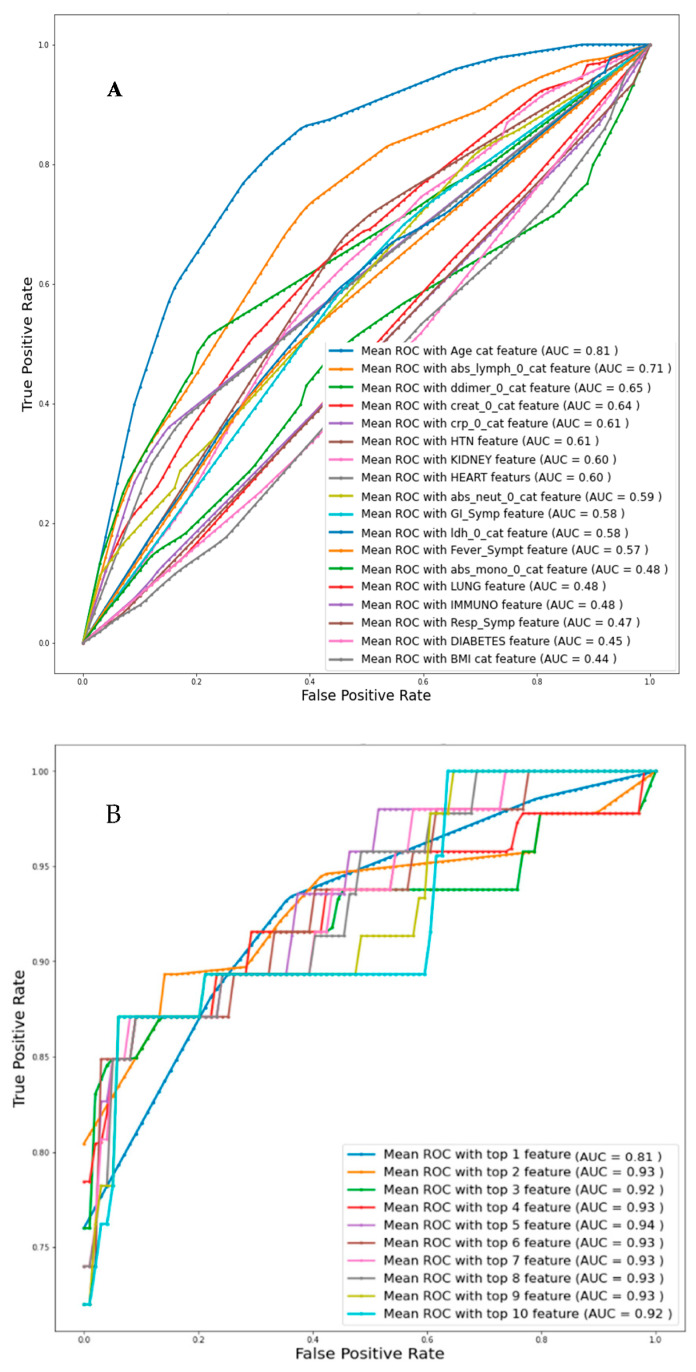
Comparison of the receive operating characteristic (ROC) plots for (**A**) individual feature (Imputation-Mice, Classifier-Logistic regression), (**B**) top-ranked 1 up to 10 features (Imputation-Mice, Classifier-Logistic regression).

Univariate logistic regression analysis with individual features was used to identify the independent variables related to death, and then the Top-1, Top-2, and up to Top-10 features were identified based on AUC values for Day-0 data from Dataset-1 as can be seen from Table 4A. Figure 3A represents the ROC curve for all the features individually and the corresponding AUC value is mentioned. Figure 3B presents the ROC curve for the combinations of the top ranked features and it is found that the combination of top ranked five features had a maximum AUC of 0.94. Overall accuracies and weighted average performance matrices for different models using Top-1 to 10 features individually and in combination using five-fold cross-validation for the logistic regression classifier are shown in Table 4A,B respectively. Each of the cases is reported with the confusion matrices so that the false positive and negative cases can be reported.

The top-ranked five independent variables: Age, Lymphocyte count, D-dimer, CRP, and Creatinine (in short ALDCC) have exhibited the best performance. Therefore, in the rest of the study, these five variables were used for nomogram creation and scoring technique development and validation.

### 3.2. Nomogram-Based Scoring System

The top-ranked five biomarkers were found to be statistically significant using an ML-based classifier to develop a multivariate logistic regression-based nomogram for predicting mortality. Table 5 shows the multivariable logistic regression analysis of the correlation between linear death prediction and biomarkers with the regression coefficient, *z*-value, standard error, and statistical significance, as well as the 95% confidence interval. The ratio of the regression coefficient to its standard error is known as the *z*-value. In logistic regression, the *z*-value typically identifies strong and weak contributors, with a high *z*-value indicating a strong relationship between the dependent and independent variables and a *z*-value near to zero indicating a weak relationship. Creatinine is not a particularly powerful predictor from the five variables, while age and Lymphocyte count are. A null hypothesis for a certain regression coefficient can be used to calculate the *p*-value, which is used to identify the significance of a specific *X*-variable in relation to the *Y*-variable. The *X*-variables with a strong correlation to the *Y*-variables are those with a *p* < 0.05. The *p*-value also shows that Creatinine is only weakly connected to the *Y*-variable. However, the logistic regression classifier in Table 4B shows that the five variable model outperforms the four variable models. As a consequence, no variable from these five variables was deleted when developing the nomogram.

**Figure 4:**
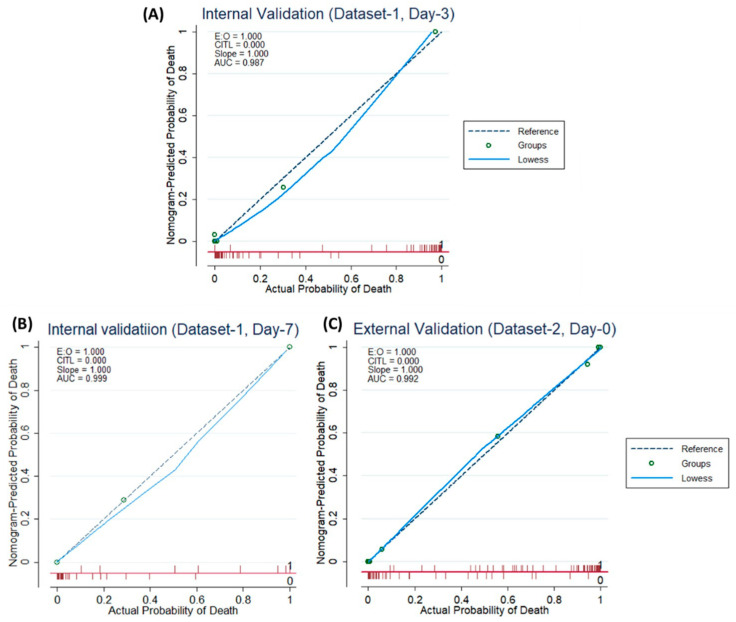
Internal Validation Curve (**A**,**B**) and the External validation (**C**).

According to Figure 4A–C, both for internal and external validation, the calibration curve matches closely with the diagonal line which is representative of the ideal model. Supplementary Figure S3 shows that the net gain of single Age and Lymphocyte count predictor models are positive, once the threshold of 0.85 is reached. This means that they both contributed the most to the prediction of the results. Interestingly, the complete model showed the best results, which also reinforced the need to combine the model with five predictors.

**Figure 5:**
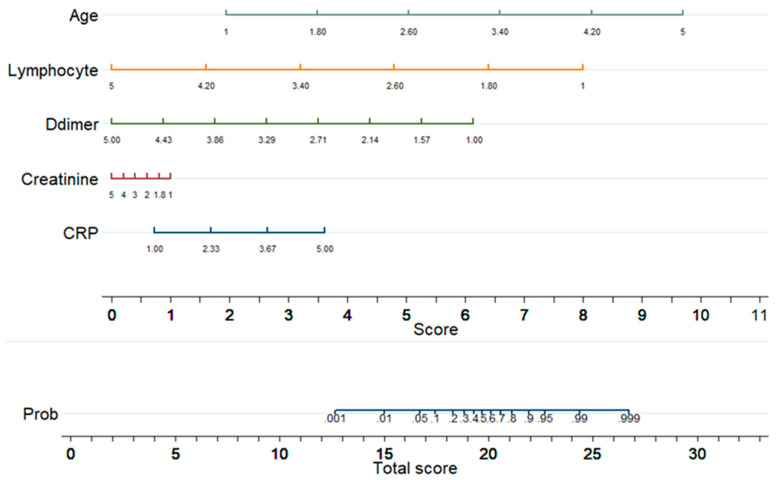
Developed Nomogram.

Figure 5 shows a nomogram with eight rows with different colors so that they are distinguishable, with rows 1–5 representing independent variables. Each variable was assigned a score by drawing a downward vertical line from the value on the variable axis to the ‘points’ axis using patient data. The score (row 6) corresponds to the points of the five variables, and the scores are added to the overall score (row 8). A line could then be drawn from the ‘Total Score’ axis to the ‘Prob’ axis to calculate the death risk of patients (row 7). However, the mathematical equations explaining the total score, linear prediction, and death probability based on which the ALDCC score is produced can be derived using the corresponding equations found earlier in Equations (5)–(8). The ALDCC score cut-off values of 16.6 and 19.8 correspond to 5% and 50% of the probability of mortality, respectively. This can be used to categorize all patients into three groups: low, moderate, and high-risk. The death probability was less than 5%, between 5% and 50%, and more than 50% for the low-risk group (ALDCC < 16.6), moderate risk group (16.6 ≤ ALDCC ≤ 19.8), and high-risk group (ALDCC > 19.8), respectively.

Linear prediction = −0.7606855 + 1.904726 × age − 1.964625 × lymphocyte count − 1.508334 × d-dimer + 0.709297 × CRP + 0.2467726 × creatinine(10)

Death probability = 1/(1 + exp (−Linear Prediction))(11)

### 3.3. Performance Evaluation of the ALDCC Scoring Technique

**Figure 6:**
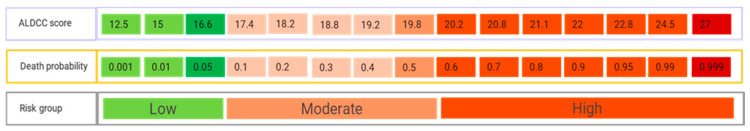
ALDCC score from nomogram and corresponding death probability of COVID-19 and non-COVID-19 patients where ALDCC score ≤ 16.6 and death probability ≤ 5% are shown for low risk group and ALDCC score > 19.8 and death probability > 50% are shown for high risk group.

**Figure 7:**
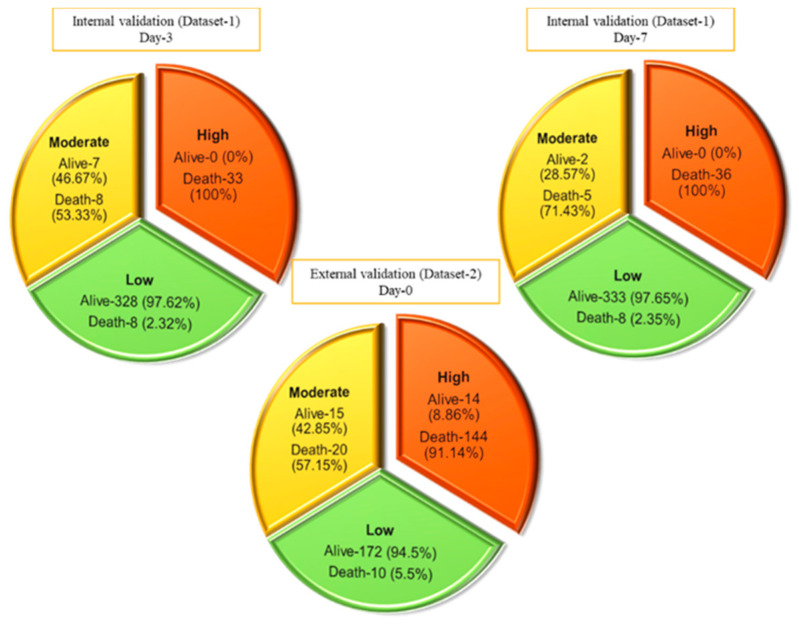
Prediction of internal and external validation with Dataset-1 and Dataset-2 using ALDCC score.

**Figure 8:**
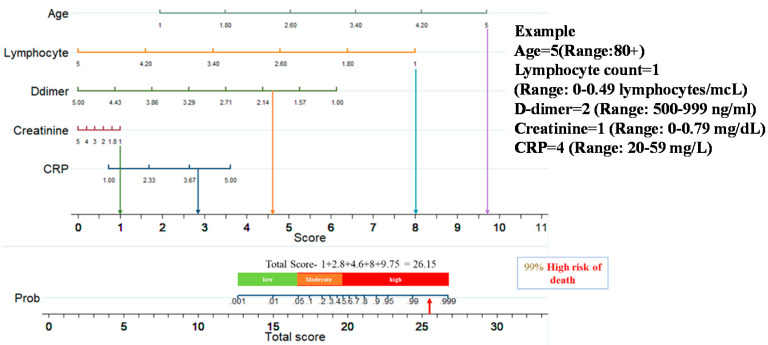
An example of nomogram-based ALDCC score to predict the probability of death of a patient from the test set (3 weeks before the actual outcome).

This nomogram-based scoring technique can be used to anticipate patient outcomes early by categorizing them into low, moderate, and high-risk categories. The thresholds for ALDCC score, which can be used to find the death probability using Equation (11), for the different categories are illustrated in Figure 6, prioritizing patients in the moderate and high-risk groups. We have categorized the patients from internal (Dataset-1, Day-3′ and Day-7 data) and external validation (Dataset-2, data on admission) into three subgroups (low, moderate, and high-risk) by associating actual outcome with the predicted outcome using the ALDCC score. For internal validation (Dataset-1 at Day-3), the proportions of death were 2.38% (8/336) (*p* < 0.001) for low-risk group, 53.33% (8/15) (*p*-value = 0.0025) for moderate-risk group and 100% (33/33) (*p* < 0.001) for high-risk group. For Dataset-1 at Day-7, the proportions of death were 2.35% (8/341) (*p* < 0.001) for low-risk group, 71.43% (5/7) (*p* < 0.001) for moderate-risk group and 100% (36/36) (*p* < 0.001) for high-risk group, while for external validation from different hospital (Dataset-2), the proportions of death were 5.5% (10/172) (*p* < 0.001) for low-risk group, 57.15% (20/35) (*p* < 0.001) for moderate-risk group and 91.14% (144/158) (*p* < 0.001) for high-risk group as shown in Figure 7. The actual death rates among the three categories were found to differ significantly (*p* < 0.001). Figure 8 illustrates a nomogram-based scoring system for a COVID-19 patient with admission variables. Individual scores for each predictor were calculated and added together to create a total score, with a death probability of 99%. This can be done as early as three weeks before the patient’s actual outcome.

## 4. Discussion

The association between the severity of the disease and the clinical evidence was explored in the current analysis. Based on the data acquired at hospital admission time, ten predictors were defined by the logistic regression algorithm as death probability predictors. Ten different classification models were trained, validated, and evaluated using this technique for the Top 1 to 10 features. The AUC and performance matrices for the top five features with the highest AUC of 0.94 were observed. A logistic regression-based nomogram was then developed utilizing these five variables. An overall score known as ALDCC has been proposed for early categorization of death severity. Moreover, the results obtained in the paper are better than in some recent similar works, as can be seen from Table 6.

Age has been identified as a primary predictor of death in earlier research on the coronavirus family, including SARS [59], Middle East respiratory disease (MERS) [60] and COVID-19 [61]. Immuno-senescence and/or various medical problems appear to make individuals more sensitive to significant COVID-19 disease with older age [55]. Increased Lymphocytes, according to Liu et al. [62], can aid in the early detection of COVID-19 disease severity. Lymphocytes, a type of immune cell, play a critical role in host defense and infection clearance. Lymphopenia, defined as a decrease in the number of blood lymphocytes, is a common biologic finding in COVID-19 patients and may play a role in disease progression and death [63]. Patients with community-acquired pneumonia have considerable immune system activation and/or immunological malfunction, leading to alterations in their levels, according to earlier studies. It has been observed [64,65] that reducing creatinine levels, due to kidney problems occurring due to COVID-19 is an indicator of COVID-19 severity and mortality. It was observed in this study that Lymphocytes and creatinine parameters were small for high-risk patients. CRP testing at the time of admission, according to Lu et al. in [66], can help to predict COVID-19-associated mortality. CRP is an acute-phase protein generated by hepatocytes in response to infection, inflammation, or tissue damage-induced cytokines from leukocytes [63,66,67,68,69,70]. This study found similar findings, with higher CRP rates estimated upon admission for COVID-19/Non-COVID individuals with high mortality risk. This indicated that these patients had severe lung inflammation or, more likely, a subsequent bacterial infection [61]. Weng et al. [55] recently indicated that individual primary predictors associated with death probability were age, Lymphocyte count, D-dimer, and CRP. A nomogram for death prediction was developed using these key predictors. In this study, a logistic regression model, using the selected five key predictors reported at admission, was used to construct a nomogram-based prognostic model that exhibits excellent calibration and discrimination in predicting the probability of death of patients with COVID-19 and non-COVID-19. An unseen external cohort was used for validation and the model also showed an outstanding performance on the external dataset. Additionally, several blood sample data obtained from patients during their hospital stay were analyzed, and the model outperformed the competition on longitudinal data. To the best of our knowledge, the AUC values for the development, internal, and external validation cohorts were 0.987, 0.999, and 0.992, respectively, which is superior to all previous nomogram-based mortality prediction methods.

Furthermore, this nomogram-derived ALDCC score provided a simple, easy-to-understand, and interpretable early warning method for stratifying and thus assisting clinical management of high-risk patients at admission. Using the ALDCC score assessed and determined at admission, all patients were grouped into three risk groups. The patients who are in the low-risk category can be isolated and handled in an isolation unit, while the isolation ward could be treated as a specialized facility with moderate-risk patients. On the other hand, patients in the high-risk community should be closely monitored and, if possible, transferred to vital care facilities or ICU for emergency treatment.

This research has scope for improvement in future. Firstly, the article suggests that clinical data on both COVID-19 and non-COVID-19 could be used to aid in the estimation of early mortality. The model can be improved much more with the help of a larger dataset. Secondly, unlike the first dataset where a limited number of parameters are present, if we have access to large features set (like Dataset-2), the machine learning model can be used to identify the best features in multi-center and multi-country data to create a more generalized model that can be used in any country.

Being able to predict the risk of mortality for patients is needed for allocating the right resources during a crisis. Indeed, very high mortality patients might not be the target for receiving the highest level of support and might need comfort care in a situation of crisis, as we have seen during the first period of the pandemic in many countries.

On the contrary, the low risk mortality patients should not be directed to demanding resources units such as ICU and can be treated outside the hospital, easing the strong pressure on the healthcare facilities. This tool might be used too in research to evaluate its ability to predict in a prospective manner the death of COVID-19 patients and refine this by including other parameters. The limitation of this kind of tool is that it takes into consideration clinical and biological parameters and does not integrate treatments, and is obviously exposed to bias.

## 5. Conclusions

In summary, the developed nomogram can be deployed for rapid and reliable mortality prediction of patients with both COVID-19 and non-COVID-19, based on multiple risk factors, such as Age, Lymphocyte count, D-dimer, CRP, and Creatinine. The model can predict the patient’s prognosis with a high accuracy, well in advance of the actual clinical outcomes. As a result, the use of ALDCC can assist physicians in developing an effective and optimized patient management strategy without overloading healthcare resources, as well as minimizing death, through an increased and expected response. The authors have also created a webpage as App [71] to assist healthcare personnel in predicting early mortality using the developed model and easily accessible ALDCC scoring results (Supplementary Figure S4). We hope to improve the model’s performance even more with the help of a larger dataset comprising data from other centers and countries.

## Figures and Tables

**Table 1:** Description of different variables in the Dataset-1.

Variable/Features	Description
COVID	COVID status (tested positive prior to enrollment or during hospitalization): 0 = negative, 1 = positive
Age	Age: 1 = 20–34, 2 = 36–49, 3 = 50–64, 4 = 65–79, 5 = 80+
BMI	Body mass index: 0 = <18.5 (underweight), 1 = 18.5–24.9 (normal), 2 = 25.0–29.9 (overweight), 3 = 30.0–39.9 (obese), 4 = ≥40 (severely obese), 5 = Unknown
Heart	Pre-existing heart disease—HEART-(coronary artery disease, congestive heart failure, valvular disease): 0 = No, 1 = Yes
Lung	Pre-existing lung disease—LUNG-(asthma, COPD, requiring home O, any chronic lung condition): 0 = No, 1 = Yes
Kidney	Pre-existing kidney disease—KIDNEY-(chronic kidney disease, baseline creatinine > 1.5, ESRD), 0 = No, 1 = Yes
Diabetes	Pre-existing diabetes—DIABETES- (pre-diabetes, insulin and non-insulin dependent diabetes): 0 = No, 1 = Yes
HTN	Pre-existing hypertension—HTN: 0 = No, 1 = Yes
Immunocompromised	Pre-existing immunocompromised condition—IMMUNO (active cancer, chemotherapy, transplant, immunosuppressant agents, aspenic): 0 = No, 1 = Yes
Resp_Symp	Respiratory symptoms—Symp_Resp (sore throat, congestion, productive or dry cough, shortness of breath or hypoxia, or chest pain): 0 = No, 1 = Yes
Fever_Sympt	Febrile symptom
GI_Symp	Any GI-related symptoms at presentation (abdominal pain, nausea, vomiting, diarrhea)
abs_neut	Absolute neutrophil count: 1 = 0–0.99, 2 = 1.0–3.99, 3 = 4.0–7.99, 4 = 8.0–11.99, 5 = 12+
abs_lymph	Absolute lymphocyte count Day-0: 1 = 0–0.49, 2 = 0.50–0.99, 3 = 1.00–1.49, 4 = 1.50–1.99, 5 = 2+
abs_mono	Absolute monocyte Day-0: 1 = 0–0.24, 2 = 0.25–0.49, 3 = 0.50–0.74, 4 = 0.75–0.99, 5 = 1.0+
Creatinine	Creatinine: 1 = 0–0.79, 2 = 0.80–1.19, 3 = 1.20–1.79, 4 = 1.80–2.99, 5 = 3+
CRP	C-reactive protein: 1 = 0–19.9, 2 = 20–59.0, 3 = 60–99.9, 4 = 100–179, 5 = 180+
D-dimer	D-dimer: 1 = 0–499, 2 = 500–999, 3 = 1000–1999, 4 = 2000–3999, 5 = 4000+
LDH	Lactate dehydrogenase: 1 = 0–200, 2 = 200–299, 3 = 300–399, 4 = 400–499, 5 = 500+
Outcome	Outcome at 28 days: 1 = Death within 28 days; 2 = Intubated, ventilated, survived to 28 days; 3 = Non-invasive ventilation or high-flow nasal cannula; 4 = Hospitalized, supplementary O_2_ required; 5 = Hospitalized, no supplementary O_2_ required; 6 = Not hospitalized.Note: this study used ‘1′ for dead patients and ‘0′ for survived patients, which are created from, class 2–6.

**Table 2:** Statistical Analysis of the Characteristic of the subjects’ data for (**A**) Dataset-1 and (**B**) Dataset-2.

**(A)**						
**Features**	**Survived (%)**	**Death (%)**	**Total**	**Method**	**Statistic**	***p*-Value**
**Age (Year)**	335 (missing-0)	49 (missing-0)	384 (missing-0)	Chi-square test	*X*^2^ = 13.45	<0.0001
1 (<34)	36 (10.75%)	0 (0%)	36 (9.38%)			
2 (34–49)	72 (21.49%)	1 (2.04%)	73 (19%)			
3 (50–64)	105 (31.34%)	6 (12.24%)	111 (28.9%)			
4 (65–79)	82 (24.48%)	17 (34.69%)	99 (25.78%)			
5 (above 80)	40 (11.94%)	25 (51.02%)	65 (16.94%)			
**Lymphocyte count (lymphocyte/mcL)**	332 (missing-3)	47 (missing-2)	379 (missing-5)	Chi-square test	*X*^2^ = 12.34	<0.0001
1 (0–0.49)	38 (11.45%)	16 (34.04%)	54 (14.24%)			
2 (0.50–0.99)	111 (33.43%)	22 (46.81%)	133 (35.09%)			
3 (1.00–1.49)	103 (31.02%)	5 (10.64%)	108 (28.5%)			
4 (1.50–1.99)	52 (15.66%)	3 (6.38%)	55 (14.51%)			
5 (above 2)	28 (8.43%)	1 (2.13%)	29 (7.66%)			
**D-dimer (ng/mL)**	312 (missing-23)	46 (missing-3)	358 (missing-26)	Chi-square test	*X*^2^ = 6.7	<0.0001
1 (0–499)	50 (16.03%)	1 (2.17%)	51 (14.24%)			
2 (500–999)	107 (34.29%)	12 (26.09%)	119 (33.24%)			
3 (1000–1999)	93 (29.81%)	14 (30.43%)	107 (29.88%)			
4 (2000–3999)	34 (10.90%)	9 (19.57%)	43 (12.08%)			
5 (above 4000)	28 (8.97%)	10 (21.74%)	38 (10.56%)			
**Creatinine (mg/dL)**	334 (missing-1)	47 (missing-2)	381 (missing-3)	Chi-square test	*X*^2^ = 11.65	<0.0001
1 (0–0.79)	113 (33.83%)	11 (23.40%)	124 (32.54%)			
2 (0.80–1.19)	155 (46.41%)	13 (27.66%)	168 (44.09%)			
3 (1.20–1.79)	36 (10.78%)	8 (17.02%)	44 (11.54%)			
4 (1.80–2.99)	16 (4.79%)	7 (14.89%)	23 (6.03%)			
5 (above 3)	14 (4.19%)	8 (17.02%)	22 (5.8%)			
**CRP (mg/L)**	321 (missing-14)	45 (missing-4)	366 (missing-18)	Chi-square test	*X*^2^ = 7.86	<0.0001
1 (0–19.9)	68 (21.18%)	4 (8.89%)	72 (19.67%)			
2 (20–59)	60 (18.69%)	5 (11.11%)	65 (17.76%)			
3 (60–99.9)	64 (19.94%)	8 (17.78%)	72 (19.67%)			
4 (100–179)	69 (21.50%)	17 (37.78%)	86 (23.5%)			
5 (above 180)	60 (18.69%)	11 (24.44%)	71 (19.4%)			
**(B)**						
**Features**	**Survived**	**Death**	**Total**	**Method**	**Statistic**	***p*-Value**
	**Frequency (%)**	**Frequency (%)**				
**Age (Year)**	201 (missing-0)	174 (missing-0)	375 (missing-0)	Chi-square test	*X*^2^ = 1.89	<0.0001
1 (<34)	36 (17.91%)	2 (1.15%)	38 (10.13%)			
2 (34–49)	59 (29.35%)	7 (4.02%)	66 (17.60%)			
3 (50–64)	63 (31.34%)	47 (27.01%)	110 (29.33%)			
4 (65–79)	39 (19.40%)	86 (49.43%)	125 (33.33%)			
5 (above 80)	4 (1.99%)	32 (18.39%)	36 (9.60%)			
**Lymphocyte count (lymphocytes/mcL)**	194 (missing-7)	162 (missing-12)	356 (missing-19)	Chi-square test	*X*^2^ = 8.23	<0.0001
1 (0–0.49)	8 (4.12%)	66 (40.74%)	74 (20.79%)			
2 (0.50–0.99)	81 (41.75%)	78 (48.15%)	159 (44.66%)			
3 (1.00–1.49)	61 (31.44%)	15 (9.26%)	76 (21.35%)			
4 (1.50–1.99)	30 (15.46%)	2 (1.23%)	32 (8.99%)			
5 (above 2)	14 (14%)	1 (0.62%)	15 (4.21%)			
**D-dimer (ng/mL)**	182 (missing-19)	160 (missing-14)	342 (missing-33)	Chi-square test	*X*^2^ = 7.22	<0.0001
1 (0–499)	79 (43.41%)	7 (4.38%)	86 (25.15%)			
2 (500–999)	49 (26.92%)	16 (10%)	65 (19.01%)			
3 (1000–1999)	36 (19.78%)	19 (11.88%)	55 (16.08%)			
4 (2000–3999)	10 (5.49%)	27 (16.88%)	37 (10.82%)			
5 (above 4000)	8 (4.40%)	91 (56.88%)	99 (28.95%)			
**Creatinine (mg/dL)**	193 (missing-8)	163 (missing-11)	356 (missing-19)	Chi-square test	*X*^2^ = 11.89	<0.0001
1 (0–0.79)	134 (69.43%)	61 (37.42%)	195 (54.78%)			
2 (0.80–1.19)	44 (22.80%)	67 (41.10%)	111 (31.18%)			
3 (1.20–1.79)	11 (5.70%)	19 (11.66%)	30 (8.43%)			
4 (1.80–2.99)	0 (0%)	10 (6.13%)	10 (2.81%)			
5 (above 3)	4 (2.07%)	6 (3.68%)	10 (2.81%)			
**CRP (mg/L)**	194 (missing-7)	159 (missing-15)	353 (missing-22)	Chi-square test	*X*^2^ = 7.01	<0.0001
1 (0–19.9)	100 (51.55%)	5 (3.14%)	105 (29.75%)			
2 (20–59)	58 (29.90%)	32 (20.13%)	90 (25.50%)			
3 (60–99.9)	17 (8.76%)	30 (18.87%)	47 (13.31%)			
4 (100–179)	17 (8.76%)	52 (32.70%)	69 (19.55%)			
5 (above 180)	2 (1.03%)	40 (25.16%)	42 (11.9%)			

**Table 3:** Performance Comparison between different Machine Learning Classifiers.

Machine Learning Classifier	Weighted Average (95% Confidence Interval)				Overall Accuracy
	Precision	Sensitivity	F1-Score	Specificity	
KNN	0.88 ± 0.10	0.87 ± 0.16	0.88 ± 0.13	0.77 ± 0.08	0.88 ± 0.07
Random Forest	0.89 ± 0.11	0.88 ± 0.12	0.88 ± 0.12	0.77 ± 0.11	0.89 ± 0.05
XGBoost	0.87 ± 0.64	0.87 ± 0.42	0.86 ± 0.7	0.87 ± 0.2	0.87 ± 0.11
SVM	0.86 ± 0.03	0.85 ± 0.13	86 ± 0.03	0.86 ± 0.5	0.86 ± 0.07
Extra-tree	0.90 ± 0.024	0.89 ± 0.015	0.90 ± 0.01	0.90 ± 0.025	0.89 ± 0.012
**Logistic Regression**	0.92 ± 0.03	0.91 ± 0.03	0.92 ± 0.03	0.78 ± 0.04	0.91 ± 0.03

**Table 4:** Comparison of the average performance matrix and confusion matrix from five-fold cross-validation for (**A**) Individual top 10 feature, (**B**) Combined top 1 to 10 features.

**(A)**									
**Features**	**AUC**	**Accuracy**	**Precision**	**Recall**	**F1 Score**	**Specificity**			
Age	0.81	0.85 ± 0.04	0.86 ± 0.03	0.85 ± 0.04	0.85 ± 0.04	0.56 ± 0.05			
Lymphocyte count	0.71	0.72 ± 0.10	0.75 ± 0.08	0.72 ± 0.06	0.72 ± 0.07	0.55 ± 0.17			
D-Dimer	0.65	0.66 ± 0.08	0.67 ± 0.13	0.66 ± 0.04	0.65 ± 0.07	0.80 ± 0.14			
Creatinine	064	0.62 ± 0.02	0.64 ± 0.02	0.62 ± 0.08	0.61 ± 0.06	0.80 ± 0.04			
CRP	0.61	0.57 ± 0.05	0.57 ± 0.05	0.57 ± 0.04	0.57 ± 0.04	0.59 ± 0.09			
HTN	0.61	0.65 ± 0.04	0.65 ± 0.04	0.65 ± 0.13	0.65 ± 0.07	0.66 ± 0.10			
Kidney	0.60	0.54 ± 0.09	0.54 ± 0.08	0.54 ± 0.11	0.54 ± 0.09	0.50 ± 0.10			
Heart	0.60	0.61 ± 0.09	0.69 ± 0.06	0.61 ± 0.06	0.56 ± 0.06	0.28 ± 0.14			
Abs Neutrophil	0.59	0.61 ± 0.011	0.62 ± 0.09)	0.61 ± 0.014	0.60 ± 0.10	0.48 ± 0.14			
GI_Symp	0.58	0.57 ± 0.03	0.57 ± 0.03	0.57 ± 0.05	0.56 ± 0.04	0.71 ± 0.03			
**(B)**									
**Features**	**Overall Accuracy**	**Weighted Performance with 95% CI**				**Confusion Matrix**			
						**Death**		**Alive**	
		**Precision**	**Recall**	**F** **1 Score**	**Specificity**	**TP**	**FP**	**FN**	**TN**
Top 1 feature	0.85 ± 0.04	0.86 ± 0.03	0.85 ± 0.04	0.85 ± 0.04	0.56 ± 0.05	25	24	34	301
Top 2 features	0.91 ± 0.03	0.92 ± 0.03	0.91 ± 0.03	0.91 ± 0.03	0.76 ± 0.04	36	13	22	313
Top 3 features	0.91 ± 0.03	0.92 ± 0.03	0.91 ± 0.03	0.91 ± 0.03	0.78 ± 0.04	37	12	22	313
Top 4 features	0.9 ± 0.03	0.91 ± 0.03	0.9 ± 0.03	0.9 ± 0.03	0.74 ± 0.04	35	14	24	311
Top 5 features	0.92 ± 0.03	0.93 ± 0.02	0.92 ± 0.03	0.93 ± 0.03	0.83 ± 0.04	40	9	20	315
Top 6 features	0.92 ± 0.03	0.93 ± 0.03	0.92 ± 0.03	0.92 ± 0.03	0.81 ± 0.04	39	10	21	314
Top 7 features	0.91 ± 0.03	0.92 ± 0.03	0.91 ± 0.03	0.91 ± 0.03	0.78 ± 0.04	37	12	23	312
Top 8 features	0.91 ± 0.03	0.92 ± 0.03	0.91 ± 0.03	0.92 ± 0.03	0.8 ± 0.04	38	11	22	313
Top 9 features	0.92 ± 0.03	0.93 ± 0.03	0.92 ± 0.03	0.92 ± 0.03	0.8 ± 0.04	38	11	20	315
Top 10 features	0.91 ± 0.03	0.92 ± 0.03	0.91 ± 0.03	0.92 ± 0.03	0.78 ± 0.04	37	12	21	314

**Table 5:** The logistic regression model.

Outcome	Coef.	Std. Err.	z	*p* > |z|	[95% Conf. Interval]	
Age	1.90473	0.49970	3.81	0.000	0.92533	2.88412
Lymphocyte count	−1.96463	0.47123	−4.17	0.000	−2.88822	−1.04103
D-Dimer	−1.50833	0.57196	−2.64	0.008	−2.62936	−0.38732
CRP	0.70930	0.44818	1.58	0.114	−0.169129	1.58772
Creatinine	−0.24677	0.40469	−0.61	0.542	−1.03995	0.54641
_cons	−0.76069	2.53057	−0.30	0.764	−5.72051	4.19914

**Table 6:** Performance comparison with similar recent works.

Paper	Patient Count	Patient Condition	Methodology in the Paper	Reported Performance
Weng et al. [55]	301 patients	Confirmed COVID-19	A nomogram was constructed to predict the death probability of COVID-19 patients. Age, neutrophil-to-lymphocyte ratio, d-dimer and C-reactive protein obtained on admission were identified as predictors of mortality for COVID-19 patients by LASSO.	The nomogram demonstrated good calibration and discrimination with the area under the curve (AUC) of 0.921 and 0.975 for the derivation and validation cohort, respectively.
Jianfeng Xie et al. [56]	299 patients and external validation with 145 patient	Confirmed COVID-19	Logistic regression analysis with the outcome variable defined as mortality.	Discrimination of the model was excellent in both internal (c = 0.89) and external (c = 0.98) validation.
Yan et al. [18]	485 patients	Confirmed COVID-19	Logistic regression-based machine learning tools selected three biomarkers that predict the mortality of individual patients	90% accuracy for mortality prediction.
Zhang et al. [57]	82 Patients	Confirmed COVID-19	The association between the different clinical variables and the time from initial symptom to death was evaluated using Spearman’s rank correlation coefficient.	Most of the death cases were male (65.9%). More than half of dead patients were older than 60 years (80.5%) and the median age was 72.5 years.
Youha et al. [58]	752 patients	Confirmed COVID-19	Association of scores with documented hard endpoints (ICU admission or death) were assessed using binary logistic regression.	The area under the curve was equal to 90.4%
Proposed Study	384 patients for Internal validation and 375 patients for external validation	Confirmed COVID-19 and Non-COVID-19	Different ML classifier and developed a nomogram with the best performed model.	For the internal (Day-3 and Day-7) and external validation cohort, the area under the curves (AUCs) were 0.987, 0.999, and 0.992, respectively.

## Data Availability

Datasets are publicly available in [39,40].

## Supplementary Materials

The following are available online at https://www.mdpi.com/article/10.3390/diagnostics11091582/s1, Figure S1: The number of missing fata for different features in Dataset-1. Figure S2: Heatmap of correlation among different features. Figure S3: Decision curves analysis stating the performance of the individual parameters and the developed model. Figure S4: Mortality risk prediction App.
