# Supplementary material for: Mortality Prediction Utilizing Blood Biomarkers to Predict the Severity of COVID-19 Using Machine Learning Technique
Source: Diagnostics (Basel). 2021 Aug 31;11(9):1582. doi: 10.3390/diagnostics11091582 (PMC8469072; doi:10.3390/diagnostics11091582)
Supplement: Supplementary file 1 [file diagnostics-11-01582-s001.zip › diagnostics-1312319-supplementary.pdf]

Mortality Prediction Utilizing Blood Biomarkers to Predict the Severity of COVID-19 using Machine Learning Technique

Tawsifur Rahman[1], Fajer A. Al-Ishaq[2], Fatima S. Al-Mohannadi[2], Reem S. Mubarak[2], Maryam H. Al-Hitmi[2], Khandaker Reajul Islam[1], Amith Khandaker[1], Ali Ait Hssain[3], Somaya A. Al Maadeed[4], Susu M. Zughaier[*2], Muhammad E. H. Chowdhury[*1]

Supplementary Materials:

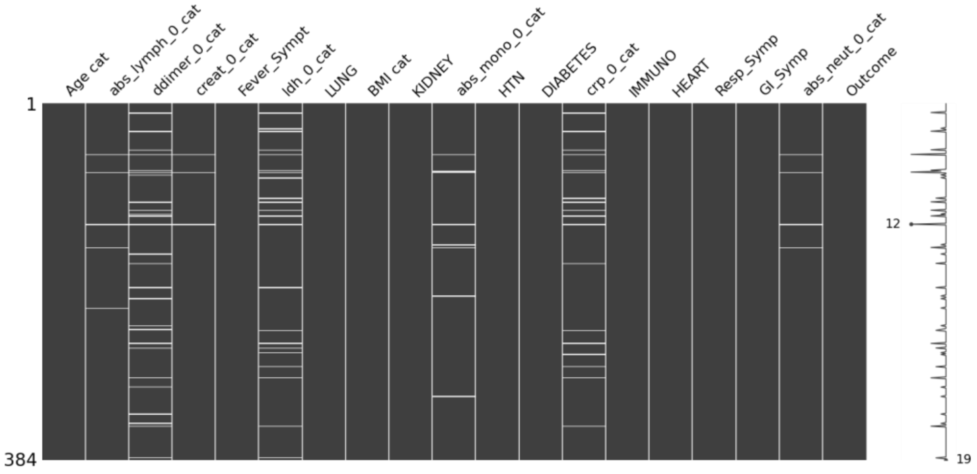

Supplementary Figure S1: The number of missing data for different features in Dataset-1.

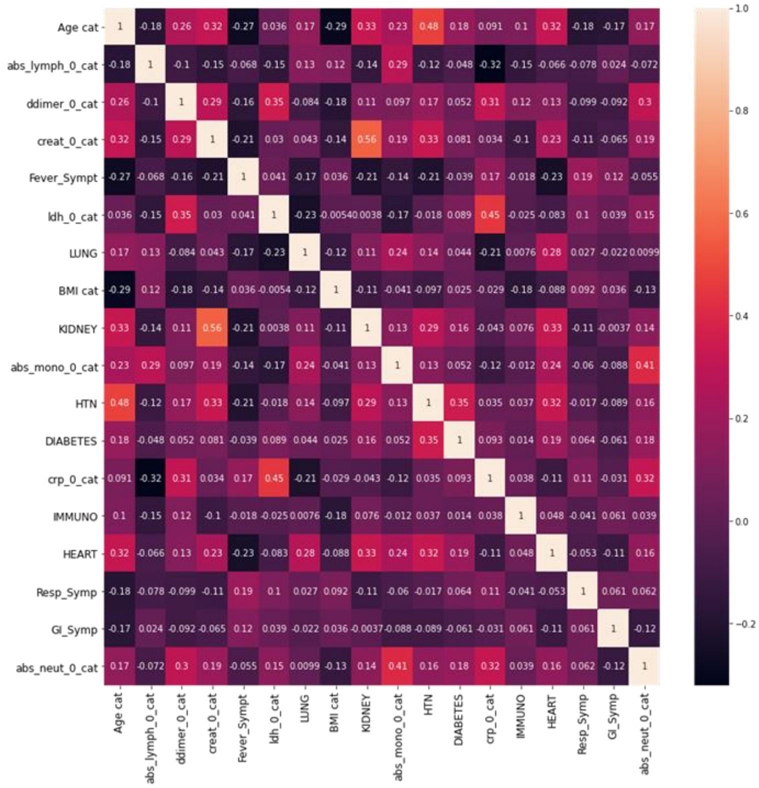

Supplementary Figure S2: Heatmap of correlation among different features.

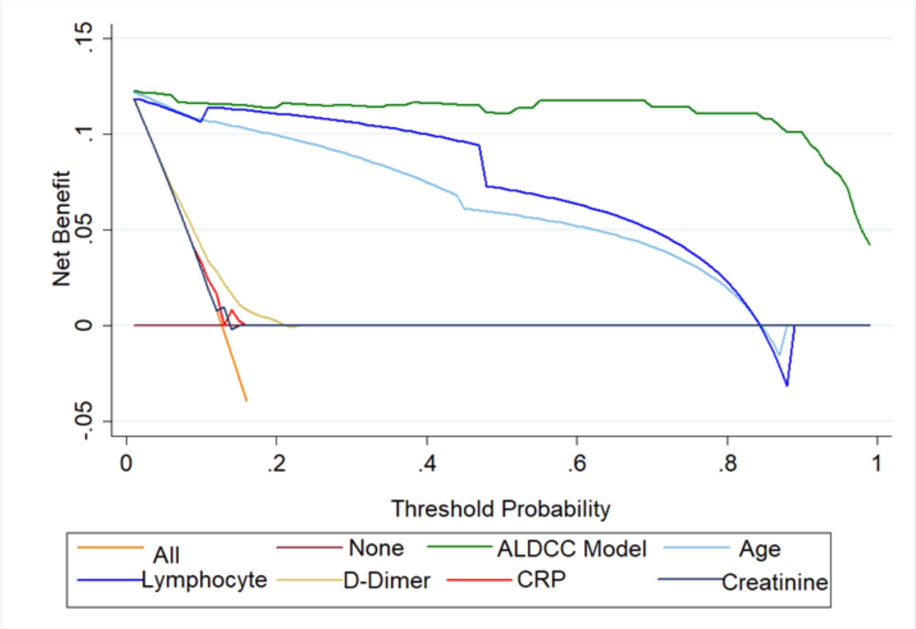

Supplementary Figure S3: Decision curves analysis stating the performance of the individual parameters and the developed model.

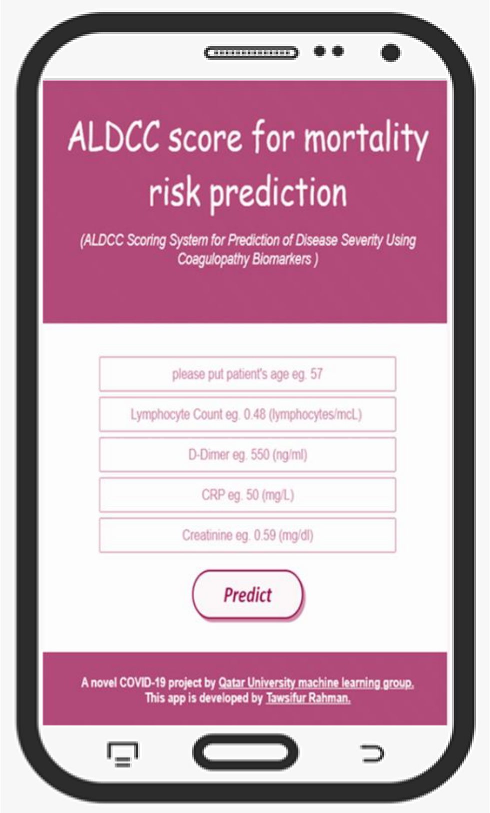

Supplementary Figure S4: Mortality risk prediction App
